# Flavan-3-ol fraction from cocoa powder promotes mitochondrial biogenesis in skeletal muscle in mice

**DOI:** 10.1186/1476-511X-13-64

**Published:** 2014-04-05

**Authors:** Naoki Watanabe, Kodai Inagawa, Masahiro Shibata, Naomi Osakabe

**Affiliations:** 1 Department of Bio-science and Engineering, Shibaura Institute of Technology, 307 Fukasaku, Munumaku, Saitama 337-8570, Japan

**Keywords:** Flavan-3-ols, Lipolysis, β-oxidation, Uncoupling protein, Mitochondria biogenesis, Mice

## Abstract

**Background:**

Numerous clinical studies have reported that ingestion of chocolate has reduced risk of metabolic syndrome. In order to elucidate the mechanism, we evaluated the influence of flavan-3-ols derived from cocoa powder on energy metabolism in mice using an indirect calorimetric method.

**Method:**

The mice were divided into two groups, and administered either distilled water or 50 mg/kg of flavan-3-ol fraction for 2 weeks. At the end of the experimental period, animals were sacrificed after blood pressure and the mean respiratory exchange ratio (RER) over 24 hours were measured.

**Results:**

The mean respiratory exchange ratio (RER) over 24 hours was reduced significantly in the flavan-3-ols group. The mean blood pressure was significantly decreased in flavan-3-ols treatment group compared with control group. The protein level of carnitine palmitoyltransferase 2 (CPT2) was increased significantly by flavan-3-ols in skeletal muscle, but not in liver. Uncoupling protein (UCP) 1 was increased significantly in brown adipose tissue by flavan-3-ols. The mitochondria copy number in gastrocnemius and soleus muscles and brown adipose tissue were increased significantly by administration of flavan-3-ol fraction.

**Conclusion:**

These results suggest that flavan-3-ols enhances lipolysis and promotes mitochondrial biogenesis. We conclude that improvement of metabolic syndrome risk factors following ingestion of chocolate may be induced, in part, by the mitochondrial biogenesis-promoting effect of flavan-3-ols.

## Background

Flavan-3-ols, a group of polyphenolic substances, are distributed in a number of plant foods such as cocoa beans, red wine, and apples. Of these foods, chocolate is known to be rich in flavan-3-ols, including the flavan 3-ol monomers, (+)-catechin and (−)-epicatechin, and oligomers, B-type procyanidins that are linked by C4-C8 bonds [1-3]. Recent meta-analyses suggested that ingestion of chocolate reduced the risk of cardiovascular diseases [4,5]. These reports showed that chocolate consumption was associated with a substantial reduction in the risk of cardiometabolic disorders such as coronary heart disease and stroke. In addition, numerous randomized, controlled trials have confirmed that chocolate, especially dark chocolate containing large amounts of flavan-3-ols, improves risk factors for the metabolic syndrome such as hypertension [6,7], vascular endothelial dysfunction [8,9], dyslipidemia [10,11], and glucose intolerance [12,13]. Several meta-analyses conducted after these clinical trials confirmed that dark chocolate rich in flavan-3-ols reduced the risk of cardiovascular disease by improving these risk factors [14-19].

On the other hand, it has been reported that the absorption rate of flavan-3-ol monomers was only about 10 - 20%, with these compounds present in blood mainly as metabolites such as conjugated forms with glucuronide and/or sulfate. In contrast, unchanged forms were nearly absent after ingestion of flavan-3-ols [20,21]. Flavan 3-ol oligomers were also shown to be poorly absorbed via the gastrointestinal tract and present in very low concentrations in the blood [22,23]. The mechanism of the multiple improvements caused by chocolate on risk factors of the metabolic syndrome has therefore remained unclear.

In order to elucidate this mechanism, we evaluated the effect of repeated supplementation of flavan-3-ols on energy metabolism using an indirect calorimetric method. We also compared the changes of uncoupling protein, lipid metabolizing enzymes and mitochondria copy number in several tissues between with or without treatment of flavan-3-ols.

## Methods

### Materials

The flavan 3-ol fraction was provided by Meiji Co., Ltd (Tokyo, Japan). The flavan- 3-ol fraction was prepared from cocoa powder by the method described in a previous report [24]. Briefly, cocoa powder was defatted with n-hexane, and then the residue was extracted with acetone. The n-butanol dissolved fraction of the extract was applied to a Diaion HP2MG column (Mitsubishi Kasei Co. Ltd., Tokyo, Japan). The fraction eluted with 80% ethanol was collected, freeze dried, and then used. The composition of this fraction was shown in Table 1. Total polyphenol was as determined by the Prussian blue method [24]. Catechins. procyanidins and xanthine derivatives were analyzed by an HPLC method [25]. Briefly, the analysis was carried out by linear gradient with 0.1% trifuloroacetic acid in CH_3_CN and 0.1% trifuloroacetic acid in H_2_O using Deverosil ODS HG-5 column (Nomura Chemical CO. Ltd., Aichi, Japan) and detected at 280 nm.

**Table 1:** Composition of flavan-3-ols fraction derived from cocoa powder

	**w/w %**
Total polyphenol^1)^	72.4
(+)-catechin^2)^	4.56
(-)-epicatechin^2)^	6.43
procyanidin B2^2)^	3.54
procyanidin B5^2)^	0.85
procyanidin C1^2)^	2.36
cinnamtannin A2^2)^	1.45
caffeine^2)^	N.D.
theobromine^2)^	N.D.

^1)^Total polyphenols was determined Prussian blue method [24].

^2)^Catechins, procyanidins and xanthine derivatives were determined by HPLC method [25].

### Animals and diets

The study was approved by the Animal Care and Use Committee of Shibaura Institute of Technology. All the animals received humane care under the guidelines of this institution.

Seven weeks old male C57BL/J mice were obtained from Clea Japan (Tokyo, Japan). The mice were kept in a room at a regulated temperature of 23–25°C and controlled lighting (12-h light and dark cycles). The basal diet was MF obtained from the Oriental Yeast, Co. Ltd., Tokyo, Japan.

### Experimental procedures

Twelve animals were fed the basal diet for 4 days and then allocated to two groups. Mice in the control group received oral intake of distilled water, while animals in the flavan-3-ols group were administrated flavan-3-ols (50 mg/kg b.w./day, p.o.) in distilled water for 2 weeks. At the end of this supplementation period, each mouse was placed into an open-circuit metabolic chamber for a 24-hour fasting period, and then VO_2_ and VCO_2_ were determined by indirect calorimetry using a small animal metabolic measurement system (MK-5000RQ Muromachi Kikai Co. Ltd, Tokyo, Japan). The system monitored VO_2_ and VCO_2_ at 3-min intervals and calculated the respiratory exchange ratio (RER). RER was calculated by following formula; RER = VCO_2_/VO_2._ The measurements of VO_2_ and VCO_2_ were converted to resting energy expenditure (REE) (kcal/day) using the Weir equation by following formula; REE = (3.941VO_2_ + 1.11VCO_2_)*1.44*60(min)*24(h). To measure spontaneous motoractivity while sedentary, the mouse was placed individually in a chamber equipped with an infrared-ray passive sensor system (MMP10, Muromachi Kikai). Measurements were performed during dark (from 19:00 to 7:00) and light (from 7:00 to 16:30) periods under fasting conditions. The blood pressure of each animal was measured by a tail-cuff method (MK-2000ST, Muromachi Kikai Tokyo Japan) before and at the end of the supplementation period. After all the measurements had been completed, the mouse was anesthetized and its blood sample was collected from the abdominal vein using heparinized syringes. Tissues samples were then collected by dissection and snap frozen in liquid nitrogen and stored at −80°C until analysis.

### Plasma biochemical assays

Plasma glucose and free fatty acid levels were determined by commercially available kits (Wako glucose CIItest and Wako NEFA C-test; Wako Chemicals, Tokyo, Japan).

### Western blotting analysis

Each tissue was homogenized in micro tubes with lysis buffer (CelLytic™ MT cell lysis Reagent: SIGMA-Aldrich, Japan) containing protease inhibitor (SIGMA-Aldrich Japan) and 0.2% SDS. The protein concentration was measured by the Bradford method. A 50 μg aliquot of protein was separated by SDS-PAGE using 4-12% Bis-Tris gel and then transferred onto polyvinylidene difluoride membranes (Life Technology, USA). The membranes were blocked with membrane blocking reagent (GE Healthcare, Buckinghamshire, UK) for 1 hour, and then incubated for 2 hours with rabbit polyclonal primary antibodies against either UCP1 (1:2500; ab1983, Abcam, Cambridge, UK), UCP2 (1:2500; ab97931, Abcam), UCP3 (1:2000; ab3477, Abcam), CPT-2 (1:500; sc-20671, Santa Cruz, Santa Cruz, USA), MCAD (1:1000; sc-98926, Santa Cruz) or α-tubulin (1:2000; ab4074, Abcam). After the primary antibody reaction, the membranes were incubated for 1 hour with the appropriate horseradish peroxidase-conjugated secondary antibody (1:100000). Immunoreactivity was detected by chemiluminescence using the ECL select™ Western Blotting Reagent (GE Healthcare, Buckinghamshire, UK). Fluorescence band images were analyzed using Just TLC (Liponics, Tokyo, Japan) analysis software. Each value was normalized relative to α-tubulin.

### Measurement of mitochondrial copy number

To measure mitochondrial DNA (mtDNA) copy number, total DNA was isolated from each organ using the QIAamp® DNA mini kit (QIAGEN Ltd., Tokyo, Japan), and 50 ng of total DNA used for real-time PCR. PCR was performed using a Step One real-time PCR system (Applied Biosystems Japan Ltd., Tokyo, Japan). The primer and probe sets used were TaqMan® Gene Expression Assay (Applied Biosystems Foster City, USA; ACTB, Mm00607939_s1; CYTB, Mm04225274; ND1, Mm04225271_g1). The buffer used in the system was TaqMan® Gene Expression Master Mix (Applied Biosystems). The PCR cycling conditions were 50°C for 2 min, 95°C for 10 min, followed by 40 cycles of 95°C for 15 s, and 60°C for 1 min. Data analysis was based on measurement of the cycle threshold (C_T_), which represents the PCR cycle count when fluorescence measurement reaches a target value. mtDNA copy number was expressed relative to nuclear DNA following amplification of the mitochondrial gene region (cytochrome b and NADH dehydrogenase 1 vs. the nuclear endogenous control region, β-actin).

### Data analysis and statistical methods

The data were expressed as the mean and standard deviation. The effect of each treatment, if any, was then compared to the control using a two-sample *t*-test. *P* values < 0.05 were considered statistically significant.

## Results

### Body weight, tissue weight, plasma biochemical parameters, and blood pressure

Table 2 shows the body weight, tissue weight, and blood pressure of the animals at the end of the supplementation period. There was no significant difference in body or tissue weight between the two test groups. The administration of flavan-3-ols caused a small but statistically insignificant increase in the levels of plasma free fatty acids and a significant decrease in plasma glucose levels compared with the control group (p < 0.05). Mean blood pressure was reduced significantly in the flavan-3-ols group compared with the control group (p < 0.05).

**Table 2:** Body and tissue weight, plasma biochemicals and mean blood pressure of mice administrated saline or 50 mg/kg flavan-3-ols for 2 weeks

	**Control**		**Flavan-3-ols**	
	**(n=6)**		**(n=6)**	
Body weight, g	22.5 ±	0.76	23.0 ±	0.26
Liver, g	0.85 ±	0.03	0.85 ±	0.04
Gastrocnemius, g	0.31 ±	0.03	0.33 ±	0.02
Soleus, g	0.02 ±	0.003	0.02 ±	0.003
Brown adipose tissue, g	0.05 ±	0.01	0.07 ±	0.07
Epidermal fat, g	0.16 ±	0.04	0.21 ±	0.02
Posterior abdominal fat, g	0.06 ±	0.01	0.06 ±	0.01
Plasma free fatty acid, mg/dl	0.67 ±	0.13	0.83 ±	0.14
Plasma glucose, mg/dl	194 ±	38.8	143 ±	33.4*
Mean blood pressure, mmHg	108 ±	5.2	96.5 ±	6.0*

Each value represents mean and SD.

Significantly difference from control; *p<0.05.

### Resting energy expenditure and activity counts

**Figure 1:**
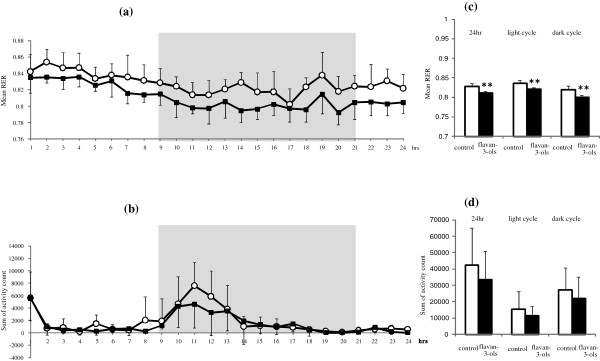
**Alterations of repeated ingestion of flavan 3-ols on whole animal energy metabolism by indirect calorimetric method.** Resting respiratory exchange ratio (RER) **(a)** and locomotor activity **(b)** were measured for 24 h after 2 weeks repeated ingestion of flavan-3-ols. Open circle indicated control group, dark square indicated flavan-3-ols treatement group. Average of RER **(c)** and total activity count **(d)** were calculated from the value during experimental period, light cycle period or dark cycle period. RER was calculated based on expired CO_2_ and O_2_ consumed (RER = VCO_2_/VO_2_). Each cage was measured for 1 min every 3 min. Gray background shading or lack thereof indicates the dark and light cycles. Values are means and SD of 6 mice. **p < 0.01 vs control group (*t* test).

Figure 1 shows the RER (a) and activity counts (b) of the animals over 24 hours at the end of the experimental period. Mean RER (c) and total activity count (d) over 24 hours that included a light cycle from 6:00 to 18:00 and dark cycle from 18:00–6:00 are also shown. As shown in Figure 1a, RER was marginally lower in the flavan-3-ols group compared with the control group throughout the measurement period, whereas there was no difference in motor-activity between the two groups (Figure 1b). There was a significant reduction in mean RER associated with flavan-3-ols treatment compared with the control group for the total duration of the experiment (Figure 1c). No difference was observed in total locomotor activity between the two groups (Figure 1d). There was not a significantly difference in REE between experimental groups.

### Western blotting analyses of β-oxidation-related enzymes in tissues

**Figure 2:**
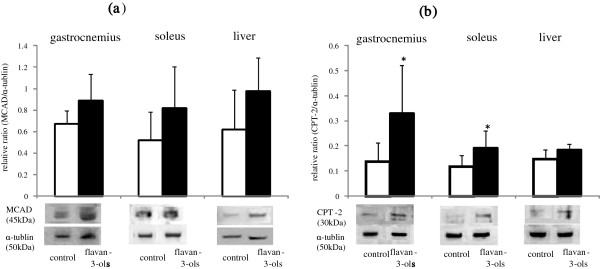
**Influence of repeated ingestion of flavan-3-ols on the enzymes in β-oxidation.** Medium-chain acyl-CoA dehydrogenase (MCAD) **(a)** and carnitine palmitoyltransferase 2 (CPT-2) **(b)** in gastrocnemius, soleus and liver were measured for 24 h after 2 weeks repeated ingestion of flavan-3-ols by western blotting analysis as described in Materials and Methods. The protein level was expressed the ratio of enzymes to α-tubulin. Values are means and SD of 6 mice. *p < 0.05 vs control group (*t* test).

As shown in Figure 2a, administration of flavan-3-ols caused a small but statistically insignificant increase MCAD protein level in the gastrocnemius and soleus muscles and liver. In contrast, CPT-2 was increased significantly in the gastrocnemius and soleus muscles following treatment with flavan-3-ols, but remained unchanged in the liver.

### Western blotting analyses f uncoupling proteins in tissues

**Figure 3:**
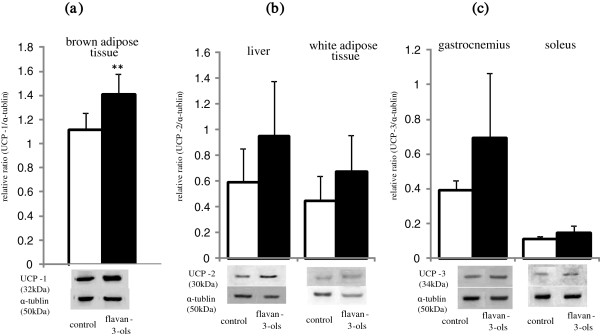
**Effect of repeated ingestion of flavan-3-ols on the uncoupling proteins.** Uncoupling protein 1 (UCP-1) in brown adipose tissue **(a)**, uncoupleing protein 2 (UCP-2) in liver **(b)** and white adipose tissue and uncoupleing protein 3 (UCP-3) **(c)** in gastrocnemius and soleus were measured for 24 h after 2 weeks repeated ingestion of flavan-3-ols by western blotting analysis as described in Materials and Methods. The protein level was expressed the ratio of enzymes to α-tubulin. Values are means and SD of 6 mice. *p < 0.05 vs control group (*t* test).

Uncoupling protein 1 (UCP-1) in brown adipose tissue was elevated significantly in the flavan-3-ols group compared with the control group (Figure 3a). As shown in Figure 3b, there was no significant difference uncoupling protein 2 (UCP-2) in the liver or white adipose tissue between the two groups. Uncoupling protein 3 (UCP-3) was increased slightly in the gastrocnemius and soleus muscles following treatment with flavan-3-ols, although these changes were not statistically significant (Figure 3c).

### Mitochondria copy number in tissues

**Figure 4:**
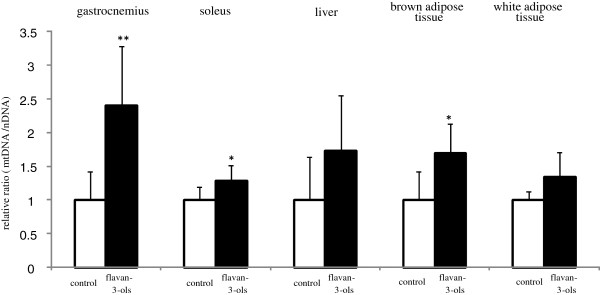
**Mitochondrial promoting activity of repeated ingestion of flavan-3-ols.** Mitochondrial DNA (mtDNA) and nuclear DNA in gastrocnemius, soleus, brown adipose tissue and white adipose tissue were measured for 24 h after 2 weeks repeated ingestion of flavan-3-ols by PCR described in Materials and Methods. mtDNA copy number was expressed relative to nuclear DNA following amplification of the mitochondrial gene region (cytochrome b and NADH dehydrogenase 1 vs. the nuclear endogenous control region, β-actin). Values are expressed as the ratio flavan-3-ols to control. Graph shows means and SD of 6 mice. *p < 0.05 vs control group (*t* test).

As shown in Figure 4, there was a significant increase in mitochondria copy number in the gastrocnemius and soleus muscles and brown adipose tissue in the flavan-3-ols group compared with the control group. However, there was no significant difference in copy number in the liver and white adipose tissue between the two experimental groups.

## Discussion

The possibility that ingestion of chocolate can reduce the risk of cardiovascular disease was reported in recent meta-analyses [4,5]. Several other meta-analyses have also demonstrated that cocoa products rich in flavan-3-ols have multiple effects on cardiovascular risk factors such as hypertension, dyslipidemia, and glucose intolerance. However, the effective component in chocolate and the mechanism of these multiple effects remains unclear. In the present study, we found that mean resting energy expenditure was reduced significantly by xanthine derivatives free flavan-3-ol fraction derived from cocoa powder (Figure 1c), and that this decrease was not affected by locomotor activity (Figure 1d). We also showed mean blood pressure and fasting blood glucose level were significantly lower in mice treated with flavan-3-ols compared with controls (Table 2). Our series of experiments on flavan-3-ols treatment showed a slight induction of MCAD in skeletal muscle a rate-controlling enzyme in β-oxidation, and a significant increase in gastrocnemius and CPT-2 in soleus muscles, a mitochondrial enzyme. In contrast, no increase in CPT-2 was observed in the liver (Figure 2b). UCP-1 in brown adipose tissue was also induced significantly by flavan-3-ols treatment, whereas UCP-2 in liver and white adipose tissue and UCP-3 in skeletal muscle remained largely unchanged (Figure 3). In addition, we confirmed that biogenesis of mitochondria was increased significantly in skeletal muscle, such as the soleus and gastrocnemius muscles (Figure 4). In contrast, no significant change was observed in mitochondria copy number in liver and white adipose tissue. Skeletal muscle is known to contribute largely to whole body energy expenditure through its mitochondrial oxidative phosphorylation and thermogenesis [26]. The promotion activity of flavan-3-ols on the skeletal muscle mitochondrial biosynthesis suggested the possibility of improvement of metabolic syndrome by additional energy expenditure.

On the other hand, several systematic reviews have confirmed that consumption of dark chocolate rich in flavan-3-ols decreases blood pressure [14-19]. The current study confirmed the reduction in blood pressure. The mechanism of this hypotensive effect has been attributed to orally-administrated flavan-3-ols being distributed in vascular endothelial cells, resulting in direct stimulation of endothelium nitric oxide synthetase [27]. As described above, flavan-3-ols other than monomers are poorly absorbed, and are unlikely to have a direct influence on endothelial cells. Further studies focusing on the circulation are therefore needed to elucidate the hypotensive mechanism of flavan-3-ols. Nevertheless, it was confirmed that the effective component in chocolate that improved metabolic syndrome risk factors was flavan-3-ols according to the results of the present study.

Numerous authors have demonstrated an increase in lipolysis following intake of polyphenols. For instance, catechins in green tea have been reported to enhance energy expenditure by inhibiting catechol O-methyl transferase [28]. These results indicate green tea does not enhance energy expenditure or lipolysis by itself. Catechins in green tea showed a significant effect on energy metabolism at a high level of blood catecholamines, such as after either caffeine ingestion or during exercise [29-33]. Chocolate also contains caffeine found in green tea. An intervention trial of caffeine demonstrated that they may contribute to alterations in energy metabolism [34]. There was limited information about the interaction between theobromine and polyphenols in cocoa. Etherton et al. reported that the xanthine derivatives unlikely to have significant impact on the reducing activity of LDL oxidative susceptibility of cocoa in randomized control study using theobromine capsule [35]. As the present study investigated xanthin drivatives-free fraction of flavan-3-ol derived from cocoa powder, the results suggest that flavan-3-ols enhance fat oxidation independent of prolonged increases in blood catecholamines levels induced by xanthine derivatives. Further evidence for this ability of polyphenols to directly modify energy metabolism was provided in a recent study by Goto et al., which showed repeated ingestion of tiliroside, a glycosidic flavonoid, reduced RER and elevated AMP-activated protein kinase in both the liver and skeletal muscle [36]. It has also been shown in mice that RER is reduced significantly by ingestion of coffee polyphenols [37].

Several reports have suggested that polyphenols inhibit glucose and fat absorption and/or digestion by inhibiting gastrointestinal enzymes [38,39]. Similarly, flavan-3-ols derived from cocoa powder have been demonstrated to inhibit dietary fat absorption [40,41]. In these studies, it was suggested that at least 300 to 800 mg/kg flavan-3-ol fraction was needed to inhibition of glucose or fat absorption and/or digestion. According to these previous results, it was unlikely suggested that the administration of 50 mg/kg flavan-3-ol fraction had a significant influence on digestion and absorption of carbohydrate or fat.

We observed an elevation in mitochondria copy number in skeletal muscle following supplementation of flavan-3-ols. A similar increase in mitochondrial biogenesis in skeletal muscle was also reported in patients with type 2 diabetes or heart failure following ingestion of cocoa [42]. According to the previous reports, peroxisome proliferator-activated receptor γ coactivator α (PGC-1α) is recognized as a master regulator of mitochondrial biogenesis [43-45] by activating respiratory chain and fatty acid oxidation genes, increasing mitochondrial number, and enhancing mitochondrial respiratory capacity. It has been shown that PGC-1α exerts these effects through direct or coactivation with peroxisome proliferator-activated receptors (PPARs), estrogen-related receptors (ERRs), and nuclear respiratory factors (NRFs). Transcription factors such as myocyte enhancer factor 2 (MEF2), forkhead box class-O (FoxO1), activating transcription factor 2 (ATF2), and cAMP response element-binding protein (CREB) enhance PGC-1α transcription during physiological stress such as exercise, cold, fasting, and increased cytokine production [46]. For UCP gene expression, PGC-1α interacts with different nuclear hormone receptors depending on the stimuli, with PPAR*γ* being one of the key transcription factors. For activation of fatty acid oxidation enzymes, PGC-1α uses different transcription factors such as PPARα and estrogen-related receptors (ERRs), that are expressed at high levels in brown adiposities [47]. In the present study, we observed an elevation in mitochondrial copy number, and induction of UCPs and β-oxidation enzymes in several tissues. These results suggested that ingestion of flavan-3-ols augmented PGC-1α transcription.

## Conclusions

This study suggested repeated ingestion of flavan-3-ols derived from cocoa was enhanced lipolysis and promoted mitochondrial biogenesis. These effects may contribute to the improvement in metabolic syndrome risk factors demonstrated in several meta-analyses.

## Abbreviations

RER: Respiratory exchange ratio; VO2: Rate of O_2_ uptake by lungs; VCO2: Rate of CO_2_ output or elimination by the lungs; REE: Resting energy expenditure; CPT2: Carnitine palmitoyltransferase; MCAD: Medium-chain acyl-CoA dehydrogenase; UCP: Uncoupling protein; NE: Norepinephrine; PGC-1α: Peroxisome proliferator-activated receptor γ coactivator α; PPARs: Peroxisome proliferator-activated receptors; ERRs: Estrogen-related receptors; MEF2: Myocyte enhancer factor 2; FoxO1: Forkhead box class-O; ATF2: Activating transcription factor 2; CREB: cAMP response element-binding protein.

## Competing interest

The authors declare that there are no competing of interest.

## Authors’ contributions

NW and KI carried out all the experiments. NW performed the data analysis and created the figures. NO and MS contributed to the design of the study. NO designed and wrote the manuscript and contributed to the final version. All authors contributed to and have approved the final manuscript.
